# Analysis of crude wastewater from two treatment plants in South Wales for 35 new psychoactive substances and cocaine, and cannabis

**DOI:** 10.1038/s41598-024-70378-7

**Published:** 2024-08-29

**Authors:** Bethan Davies, Richard Paul, David Osselton, Timothy Woolley

**Affiliations:** 1 Bournemouth University, Fern Barrow, Poole, BH12 5BB UK; 2 Inuvi Diagnostics Ltd, Churcham Business Park, Gloucester, GL28AX UK

**Keywords:** Wastewater, LC-ToF-MS, New psychoactive substances, Solid phase extraction, SWATH, Biochemistry, Toxicology

## Abstract

This study investigates the presence of new psychoactive substances (NPS) and their metabolites in two wastewater treatment plants (WWTPs) situated in South Wales, UK (WWTP-1 and WWTP-2). Analysis was conducted for 35 NPS and metabolites, along with the inclusion of benzoylecgonine (main cocaine metabolite) and cannabis, the most detected illicit substances. Benzoylecgonine was identified as the predominant substance in both WWTPs. Epidemiological calculations revealed the average population consumption of cocaine to be 3.88 mg/d/1000 inhabitants around WWTP-1 and 1.97 mg/d/1000 inhabitants for WWTP-2. The removal efficiency of benzoylecgonine across both WWTPs was observed at an average of 73%. Subsequent qualitative analyses on randomly selected wastewater samples detected medicinal compounds including buprenorphine, methadone, and codeine in both WWTPs. An additional experiment employing enzymatic hydrolysis revealed the presence of morphine, an increased presence of codeine, and 11-Nor-9-Carboxy-THC (THC-COOH) post-hydrolysis. These findings underscore the significant presence of illicit substances and medicinal compounds in wastewater systems with the absence of NPS within the South Wales area, highlighting the necessity for enhanced monitoring and treatment strategies to address public health and environmental concerns.

## Introduction

Over the past two decades, there has been a significant surge in interest regarding the utilisation of wastewater analysis for socioeconomic evaluations of drug consumption within populations. Illicit drugs and their metabolites have emerged as prevalent wastewater contaminants, posing potential yet poorly understood risks to aquatic ecosystems^1^. The emergence of new psychoactive substances (NPS), such as synthetic cannabinoids, cathinones, and benzodiazepines, has expanded the scope of wastewater testing due to their unpredictable nature and environmental impact as stated by Gent et al.^2^ and re-instated by Castiglioni et al.^3^.

The European Monitoring Centre for Drugs and Drug Addiction (EMCDDA) has spearheaded efforts to monitor illicit substances and NPS across Europe, including the UK. In a 2022 publication, the EMCDDA reported monitoring 880 NPS varieties, while approximately 83.4 million adults aged 15–64 within the European Union have been identified as consumers of illicit substances^4^. The dynamic characteristics of NPS compounds present challenges in their detection and identification within environmental samples, complicating efforts to gauge population-level drug use based on wastewater analysis^5^.

With increasing public and environmental health concerns, wastewater treatment plant (WWTP) operators are facing growing pressure to mitigate the presence of illicit substances in processed wastewater, this was illustrated by Diamind et al.^6^ and confirmed by Zhao et al.^7^. Existing research suggests that current treatment methods are insufficient in removing illicit substances effectively, as traces of these compounds persist in wastewater effluent, leading to continued environmental contamination post-treatment which was explained by Deng et al.^8^ and Yadav et al.^9^.

Despite extensive global studies on illicit substance detection in WWTPs, there remains a notable gap in research regarding drug detection specifically within WWTPs in Wales. Our study addresses this gap by investigating two WWTPs in the South Wales region, targeting the analysis of 35 NPS compounds/metabolites alongside commonly misused drug substances. Through this investigation, we aim to contribute to the understanding of drug presence in wastewater systems, emphasising the relevance of our research goals in addressing identified knowledge gaps and highlighting the novelty of our approach within the context of wastewater analysis. Despite numerous studies conducted globally on the detection of illicit substances in wastewater treatment plants (WWTPs), there has been a notable absence of research specifically focusing on drug detection within WWTPs in Wales. This gap suggests a need for localized studies to better understand the prevalence and impact of drug use on the local environment and population health. Previous research has suggested that current wastewater treatment methods may be ineffective in completely removing illicit substances, leading to traces of these compounds persisting in wastewater effluent. This gap highlights a need for improved treatment strategies to mitigate the release of illicit substances into the environment post-treatment.

By addressing these knowledge gaps, the paper aims to contribute new insights into the detection and behaviour of illicit substances and NPS within wastewater systems, particularly within the context of Wales.

## Materials and reagents

### Wastewater treatment plants

Two WWTPs in Wales, UK were monitored over a month in 2020. The samples were collected from Friday to Monday over 4 weekends. Wastewater from both WWTPs was collected at the same time and on the same weekends to compare the two locations. There is a difference between the capacity and population coverage between both WWTPs. WWTP-1 covers an estimated population of 930,624 and WWTP-2 covers an estimated population of 301,443.

### Sample collection

During the monitoring campaign, 24-h composite samples were collected from the influent wastewater from both WWTP-1 and WWTP-2. Grab samples from the post-treated effluent samples were also collected to establish whether the cleaning process was adequate and whether the compounds detected in the influent were not present in the effluent. Composite samples were obtained using an autosampler (Aquamatic Aqualcell P2-Compact) located at both WWTPs. The autosampler was programmed to collect a 1-L composite by averaging 10 mL every 15 min. One autosampler was installed at each WWTP and the samples were collected in polypropylene bottles. Grab samples collected at both WWTPs were also collected in 1-L polypropylene bottles.

### Analytical methods

The target analytes and deuterated internal standards were procured from either Chiron (Norway) or Merck (Darmstadt, Germany) at concentrations of 1 mg/mL or 100 µg/mL in methanol (MeOH) or acetonitrile (AcN). Further dilutions and preparation of working standard mixtures, with concentrations ranging between 20 and 1000 ng/L, were carried out using HPLC-grade methanol. HPLC-grade acetonitrile, methanol, and formic acid were sourced from Rathburn Chemicals (Walkerburn, UK), while ammonium acetate was obtained from Merck (Darmstadt, Germany). Ultrapure water was obtained by purifying tap water using an ELIX Millipore water purifier from Millipore (Darmstadt, Germany). Oasis HLB SPE cartridges (500mg, 6cc) were procured from Waters (New Bedford, MA, USA). Additional information regarding the analytical methods associated with this method can be found in^10^.

### Sample preparation and solid phase extraction

The samples were kept frozen until analysis and allowed to thaw in a refrigerator maintained at a temperature between 3 and 8 °C for one day.

All samples underwent solid-phase extraction (SPE) using a standardised procedure applied to all analysed substances. This SPE procedure was adapted from the protocol developed by van Nuijs et al.^11^. The method employed universal, polymeric reverse-phase SPE cartridges, specifically the Waters HLB 500 mg, 6 cc cartridges from New Bedford, MA, USA.

In detail, each sample (25 mL) was spiked with 100 µL of a mixed internal standard solution at a concentration of 50 ng/mL. The SPE cartridges were conditioned with 6 mL of methanol followed by 6 mL of deionized water. Subsequently, the samples were passed through the cartridges under vacuum at a rate of 5 mL/min. The cartridges were then washed with 3 mL of deionized water, followed by vacuum drying for 5 min. Elution was carried out using 4 mL of methanol, followed by an additional 4 mL of methanol. The eluents were dried using a sample concentrator attached to a heating block set at 55 °C.

For reconstitution, the samples were mixed with 100 µL of HPLC-grade acetonitrile, followed by 100 µL of 5 mM ammonium acetate. Subsequently, all samples were transferred to a 96-deep well plate for analysis. Notably, the samples were not pre-filtered before undergoing SPE. Further information on sample preparation and solid phase extraction can be found in^10^.

### Instrumentation and method validation

The eluents were analysed using an AB Sciex 5600 + liquid chromatography time-of-flight mass spectrometry (LC-ToF–MS) equipped with a binary pump, column oven thermostat and an electrospray ionisation (ESI) source. Chromatographic separation of each drug was performed on a YMC-Triart Phenyl 450 bar column (12 nm, 5 µm, 100 × 3 mm) (Crawford Scientific, UK). A gradient method was developed over 9:02 min including equilibrium. The mobile phase consisted of (A) 5 mM ammonium acetate and 0.2% formic acid and (B) Methanol. The collision energy (CE) for the method was set at 25 V with a collision energy spread (CES) of 15 V allowing a CE range from 10 to 40 providing a richer MS spectrum.

Quantitative analysis was performed using Sequential Window Acquisition of all Theoretical fragmentation ion spectra (SWATH®). The first SWATH® window started at 175 m/z and the final window ended at 505 m/z in positive polarity mode with a total of 50 SWATH® windows. Each SWATH® window is 5 Da wide with a 1 Da overlap. The choice of fragmentation ion for each analyte was based on the abundance of the signal, against background noise during method development. Every compound was quantified using SWATH® acquisition in positive ionisation mode where the mass of the compound was ‘searched’ within a particular SWATH® window that corresponds to the monoisotopic mass of that compound. The compound's retention time should not differ more than 2.5% from the calibration or quality control standards^12^. Analyst® software was used for system control and Multi Quant software was used for quantitative analysis. Peakview was also utilised for qualitative analysis.

Recovery was measured using a 50 ng/mL combined methanolic standard spiked into wastewater. Oasis HLB was the SPE cartridge of choice due to the array of physiochemical compounds within this study and as previous studies suggest by Kinyua et al.^13^, Shao et al.^14^, Foppe et al.^15^, Senta et al.^16^ and Gracia-Lor et al.^17^, the optimum cartridge to use for this kind of analysis. Initial optimisation of recovery was compared to Oasis MCX cartridges, results of the optimisation illustrated that Oasis HLB and Oasis MCX recoveries were comparable, both cartridges provided recoveries of around 50%. Oasis MCX cartridges required a more extensive extraction including acidification of samples before extraction compared to Oasis HLB cartridges. The efficiency of each sorbent was determined by the response of each analyte via peak area compared to an unextracted combined methanolic standard injected. Oasis HLB provided a greater peak area response for all 37 target compounds with an average recovery of 46%.

Linearity consisted of six calibration points at the following concentrations 20, 50, 100, 250, 500 and 1000 ng/L The calibration curve was used to quantify crude and effluent wastewater samples. As it was not possible to obtain an internal standard for all 38 compounds commercially, 10 internal standards were selected to cover the whole acquisition method. A 6-point calibration (R^2^ > 0.99) from 20 ng/L to 1000 ng/L was achieved for all compounds investigated in this study. LOD was determined by assessing spiked wastewater and was calculated between 4 and 20 ng/L for all analytes. LOQ was deemed to be the lowest calibrator level used within this study which was 20 ng/L.

The intra- and inter-day accuracy and precision of the method were assessed for all compounds at three concentration levels, situated at the lower end (80 ng/L), mid-range (300 ng/L) and top end (800 ng/L) of the linear range in spiked wastewater. For all analytes, the inter-day and intra-day mean accuracy was between 77 and 100%, the intra-day precision ranged between 8 and 20%, and the inter-day precision ranged between 7 and 30%.

### Estimation of drug consumption

To normalise for the variation in the daily wastewater flow rate of both WWTPs, the daily mass loads for benzoylecgonine (mg/d) were calculated using the results provided by the LC-ToF–MS analysis (ng/L) with the wastewater flow volume entering the WWTP over 24 h. Using the equations below (Eq. 1 and Eq. 2), population-normalised drug loads (mg/d/1000 inhabitants) of benzoylecgonine were then calculated. Both equations were taken from a previous study conducted by^15^. The stability of benzoylecgonine used in the equation was provided from a previous study conducted by^16^ and the correction factor from a previous study conducted by^17^

1 $${\text{Mass Load}} = {\text{C}} \times {\text{F}}\left( {\frac{{100}}{{100 \times {\text{Stability}}}}} \right) \times {\text{}}\frac{1}{{1.0 \times 10^{6} }}$$

Mass load refers to the target analyte’s calculated mass load (mg/d) C equals the concentration of the target analyte (ng/L), F equals the flow rate per day of wastewater flowing through the plant within 24 h (L/d) and stability is a measure of stability change (%) of analyte in wastewater up to 12 h. It is important to note that the stability of illicit drugs and NPS including their metabolites can vary with the composition of collected wastewater samples, which depends on the sources of wastewater and the sampling days^18^.

Similarly, community consumption was calculated using Eq. 2^19^

2 $${\text{Consumption}}/1000\;{\text{people}} = {\text{Mass}}\;{\text{Load}} \times \left( {\frac{{1000}}{{{\text{Excretion}}}}} \right) \times ~\left( {\frac{{{\text{MWpar}}}}{{{\text{MWmet}}}}} \right) \times ~\left( {\frac{{1000}}{{{\text{population}}}}} \right)$$

Consumption is mg/d/1000 people, mass load mg/d is derived from Eq. 1, and excretion is the excretion rate (%) of the parent drug or metabolite excreted from the human body after administration^20^. Mwpar is the molecular weight of the parent compound, Mwmet is the molecular weight of the metabolite, and the population is the number of people served by the WWTPs.

Benzoylecgonine has a human excretion rate of 45% as a urinary biomarker of cocaine^21^.

## Results and discussion

The results of measured concentration for each analyte and metabolite per WWTP are presented and discussed in the following sections. Both influent and effluent wastewater were analysed to check if the WWTPs adequately removed the presence of unwanted substances.

### Illicit drugs and metabolites concentration in WWTP-1 & WWTP-2

**Figure 1:**
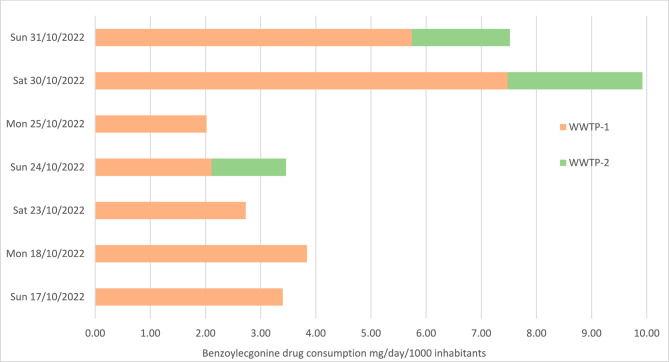
Consumption of benzoylecgonine in mg/day/1000 inhabitants in South Wales Sunday 17th October—Sunday 31st October.

**Figure 2:**
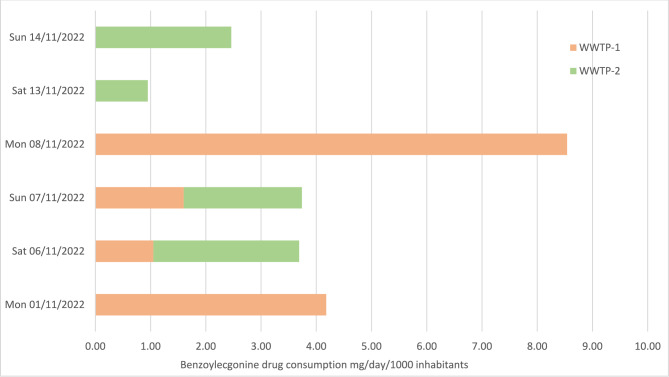
Consumption of benzoylecgonine in mg/day/1000 inhabitants in South Wales Monday 1st November—Sunday 14th November.

Table 1 summarises the measured concentrations for each target analyte. From a total of 37 compounds, only benzoylecgonine and alprazolam were detected in the crude samples. The highest benzoylecgonine concentration was detected at 6000 ng/L on 8th November 2021 and alprazolam was detected at 1300 ng/L on 25th October 2021. Each collection was carried out across the weekends when it is known that most illicit substance consumption occurs during the weekends. Figures 1 and 2 illustrate a correlation between the concentration of analyte detected and the day of the week. The highest concentration of compounds is seen on Saturdays at WWTP-1 and Mondays, for WWTP-2. All other compounds were not determined above LOQ for all samples analysed.

**Table 1:** Results of each 18 crude wastewater samples against each target compound.

Analyte	Sample number and date																	
	1	2	3	4	5	6	7	8	9	10	11	12	13	14	15	16	17	18
	17-Oct	18-Oct	23-Oct	24-Oct	24-Oct	25-Oct	30-Oct	30-Oct	31-Oct	31-Oct	01-Nov	06-Nov	06-Nov	07-Nov	07-Nov	08-Nov	13-Nov	14-Nov
25C-NBOMe	ND	ND	ND	ND	ND	ND	ND	ND	ND	ND	ND	ND	ND	ND	ND	ND	ND	ND
25I-NBOMe	ND	ND	ND	ND	ND	ND	ND	ND	ND	ND	ND	ND	ND	ND	ND	ND	ND	ND
2C-B	ND	ND	ND	ND	ND	ND	ND	ND	ND	ND	ND	ND	ND	ND	ND	ND	ND	ND
2-OXO-LSD	ND	ND	ND	ND	ND	ND	ND	ND	ND	ND	ND	ND	ND	ND	ND	ND	ND	ND
4-Methylethcathinone	ND	ND	ND	ND	ND	ND	ND	ND	ND	ND	ND	ND	ND	ND	ND	ND	ND	ND
5F-AB-PINACA	ND	ND	ND	ND	ND	ND	ND	ND	ND	ND	ND	ND	ND	ND	ND	ND	ND	ND
5F-APICA	ND	ND	ND	ND	ND	ND	ND	ND	ND	ND	ND	ND	ND	ND	ND	ND	ND	ND
5F-APINACA	ND	ND	ND	ND	ND	ND	ND	ND	ND	ND	ND	ND	ND	ND	ND	ND	ND	ND
5F-MDMB-PINACA	ND	ND	ND	ND	ND	ND	ND	ND	ND	ND	ND	ND	ND	ND	ND	ND	ND	ND
5F-PB-22	ND	ND	ND	ND	ND	ND	ND	ND	ND	ND	ND	ND	ND	ND	ND	ND	ND	ND
5-MeO-DALT	ND	ND	ND	ND	ND	ND	ND	ND	ND	ND	ND	ND	ND	ND	ND	ND	ND	ND
AB-FUBINACA	ND	ND	ND	ND	ND	ND	ND	ND	ND	ND	ND	ND	ND	ND	ND	ND	ND	ND
AB-PINACA	ND	ND	ND	ND	ND	ND	ND	ND	ND	ND	ND	ND	ND	ND	ND	ND	ND	ND
AB-PINACA Metabolite	ND	ND	ND	ND	ND	ND	ND	ND	ND	ND	ND	ND	ND	ND	ND	ND	ND	ND
Alprazolam	ND	ND	ND	ND	ND	1.3	ND	ND	ND	ND	ND	ND	ND	ND	ND	ND	ND	ND
AM2201 4-Hydroxypentyl	ND	ND	ND	ND	ND	ND	ND	ND	ND	ND	ND	ND	ND	ND	ND	ND	ND	ND
APICA 4-hydroxypentyl	ND	ND	ND	ND	ND	ND	ND	ND	ND	ND	ND	ND	ND	ND	ND	ND	ND	ND
APINACA 4-hydroxypentyl	ND	ND	ND	ND	ND	ND	ND	ND	ND	ND	ND	ND	ND	ND	ND	ND	ND	ND
APINACA 5-hydroxypentyl	ND	ND	ND	ND	ND	ND	ND	ND	ND	ND	ND	ND	ND	ND	ND	ND	ND	ND
Benzoylecgonine	3.0	3.0	2.0	1.5	2.2	1.2	4.0	1.9	3.1	1.5	2.1	0.7	4.4	1.1	3.5	6.0	1.7	4.7
Benzyl Piperazine	ND	ND	ND	ND	ND	ND	ND	ND	ND	ND	ND	ND	ND	ND	ND	ND	ND	ND
Etizolam	ND	ND	ND	ND	ND	ND	ND	ND	ND	ND	ND	ND	ND	ND	ND	ND	ND	ND
Fentanyl	ND	ND	ND	ND	ND	ND	ND	ND	ND	ND	ND	ND	ND	ND	ND	ND	ND	ND
JWH-018 Pentanoic Acid	ND	ND	ND	ND	ND	ND	ND	ND	ND	ND	ND	ND	ND	ND	ND	ND	ND	ND
LSD	ND	ND	ND	ND	ND	ND	ND	ND	ND	ND	ND	ND	ND	ND	ND	ND	ND	ND
MDMB-CHMICA	ND	ND	ND	ND	ND	ND	ND	ND	ND	ND	ND	ND	ND	ND	ND	ND	ND	ND
MDPV	ND	ND	ND	ND	ND	ND	ND	ND	ND	ND	ND	ND	ND	ND	ND	ND	ND	ND
Mephedrone	ND	ND	ND	ND	ND	ND	ND	ND	ND	ND	ND	ND	ND	ND	ND	ND	ND	ND
Methoxetamine	ND	ND	ND	ND	ND	ND	ND	ND	ND	ND	ND	ND	ND	ND	ND	ND	ND	ND
Methylone	ND	ND	ND	ND	ND	ND	ND	ND	ND	ND	ND	ND	ND	ND	ND	ND	ND	ND
Norfentanyl	ND	ND	ND	ND	ND	ND	ND	ND	ND	ND	ND	ND	ND	ND	ND	ND	ND	ND
PB-22 Carboxyindole	ND	ND	ND	ND	ND	ND	ND	ND	ND	ND	ND	ND	ND	ND	ND	ND	ND	ND
TFMPP	ND	ND	ND	ND	ND	ND	ND	ND	ND	ND	ND	ND	ND	ND	ND	ND	ND	ND
THC-COOH	ND	ND	ND	ND	ND	ND	ND	ND	ND	ND	ND	ND	ND	ND	ND	ND	ND	ND
UR-144 4-Hydroxypentyl	ND	ND	ND	ND	ND	ND	ND	ND	ND	ND	ND	ND	ND	ND	ND	ND	ND	ND
UR-144 5-Hydroxypentyl	ND	ND	ND	ND	ND	ND	ND	ND	ND	ND	ND	ND	ND	ND	ND	ND	ND	ND
UR-144 COOH	ND	ND	ND	ND	ND	ND	ND	ND	ND	ND	ND	ND	ND	ND	ND	ND	ND	ND

Due to the nature of this study being a targeted analysis rather than a non-targeted analysis, the known dynamic tendencies of NPS compounds and the amount of time taken to develop and validate a targeted analysis method, the NPS compounds in circulation could have already evolved. To determine what kind of compounds were in circulation during the time of analysis, a postcode analysis was carried out on WEDINOS. WEDINOS is an anonymous unknown substance testing service based in Wales, and their data can be filtered by postcode and date. The results shown in Table 2 illustrate what compounds were reported by WEDINOS within the relevant postcodes in the South Wales region, during the time wastewater samples were collected from the treatment plants.

**Table 2:** WEDINOS results by postcode for anonymous unknown substances submission between September 2021 and November 2021.

WWTP-1 Postcode results	WWTP-2 Postcodes results
Etizolam	CBD
4-Chlormethcathinone	Etizolam
ADB-BUTINACA	Diazepam
Amphetamine	THC
Citalopram	MDMA
Cocaine	Flubromazepam
Diazepam	Amphetamine
Eutylone	MDMB-4en-PINACA
Flubromazepam	Flualprazolam
Heroin	ADB-BUTINACA
Ketamine	Ketamine
MDMA	Cocaine
MDMB-4en-PINACA	Quetiapine
Pregabalin	
Promethazine	
THC	

The wastewater samples in this study were processed without the use of filtration. This approach mirrors a study conducted by Santana-Viera et al. in Spain^22^, where the detection of illicit substances in wastewater was explored without employing filtration before solid-phase extraction. The Spanish investigation successfully identified various illicit substances and medicinal compounds using high-resolution mass spectrometry, without the necessity of filtration. Comparable to our study in South Wales, the Spanish research also detected benzoylecgonine (with a mean concentration of 6465 ng/L-1), albeit at a notably higher concentration, likely attributable to differences in population size.

In a separate study conducted in 2020 by Pandopulos et al.^23^, the decision was made to forego filtration of the raw wastewater before analysis due to concerns regarding the potential loss of analyte concentration through filtration. By opting out of filtration, the study effectively detected novel psychoactive substances (NPS) and illicit substances, with THC-COOH registering the highest concentration at 5953 ng/L. These findings further support the rationale behind abstaining from filtration within the South Wales study during the sample preparation process. In contrast to the study, THC-COOH was not detected in our investigation. However, the higher concentration of THC-COOH compared to other detected novel psychoactive substances (NPS) within the 2020 study^23^, reaffirms that the occurrence of NPS remains lower than that of illicit substances.

The EMCDDA Drug Report 2022 published that 370 new psychoactive substances were detected in 2020 and there was a growing concern around Europe that there has been a growing crossover between illicit drugs and new psychoactive substances^4^. There has been an increase in the adulteration of low-level THC cannabis products with synthetic cannabinoids, and the production of fake opioid medications in which the main ingredient is potent benzimidazole opioids and fake Xanax and diazepam tablets containing new benzodiazepines^4^. This new trend is reflected in the WEDINOS reports. All the cases of diazepam were submitted to WEDINOS expecting the results to state diazepam but were indeed a part of the new synthetic benzodiazepines. The WEDINOS results show that cocaine is still very much a part of society and confirm the reasoning by the discovery of cocaine in all the testing WWTP samples. The reduction of the synthetic cannabinoids use is indicative of none being detected and the only synthetic cannabinoids detected are newly discovered synthetic cannabinoids (ADB-BUTINACA and MDMB-4en-PINACA) that are not included in this targeted analysis.

Di Trana et al. explored the impact of Covid-19 on drug markets in 2020. The study concluded that the COVID-19 lockdowns impacted the production, distribution, and sale of NPS and illicit substances^24^. The 2020 study stated that the production of cocaine was one of the very few substances not impacted by the COVID-19 lockdowns, again suggesting why the levels of benzoylecgonine in the WWTP samples were significant in comparison to other substances.

### Estimation of drug consumption results

In both WWTP-1 and WWTP-2, no NPS were detected. Benzoylecgonine was detected in every sample from both WWTPs, and alprazolam was discovered in one sample at WWTP-1. Figures 1 and 2 show the measured concentrations of benzoylecgonine in mg/d/1000 inhabitants from WWTP-1 and WWTP-2. WWTP-1 was found to have significantly more benzoylecgonine indicating that there is a higher proportion of cocaine use within the catchment area of WWTP-1. The highest concentration of benzoylecgonine at WWTP-1 was seen on Monday 8th November 2021 (8.54 mg/d/1000), which could account for consumption through the weekend as the level of benzoylecgonine detected on Saturday and Sunday was significantly less (1.05 mg/d/1000 and 1.60 mg/d/1000). In WWTP-2 the highest concentration of benzoylecgonine was seen on Saturday 6th of November 2021. During this period, the weekend with the highest concentration of benzoylecgonine was observed. It was the Rugby Union Autumn International in South Wales; these events are known to increase the amount of footfall within the South Wales area. In turn, this could account for the increase in concentrations observed.

Comparing the findings from the eThekwini study^25^ which also utilised the use of an LC–MS with those of the South Wales study reveals intriguing differences in drug consumption patterns between the two regions. While the eThekwini study identifies cocaine/benzoylecgonine as the dominant illicit drug consumed within the catchment area, with per capita estimates ranging from 360 to 3000 mg/day/1000 inhabitants, the South Wales study highlights the prevalence of benzoylecgonine, the main metabolite of cocaine, with average population consumption estimates of 3.88 mg/d/1000 inhabitants for WWTP-1 and 1.97 mg/d/1000 inhabitants for WWTP-2.

Furthermore, a second study was undertaken in Valencia, Spain^26^, focusing on drug consumption within the city's wastewater system. Conducted by Campo et al., the investigation examined influent wastewater samples from three distinct wastewater treatment plants (WWTPs) in Valencia. Employing solid phase extraction and ultra-high-performance liquid chromatography (UHPLC), the findings revealed the presence of illicit substances spanning 9 years, including the same timeframe as the sampling period of this South Wales study (2020). Intriguingly, akin to the outcomes of our study, the Valencia study^26^ reported a consistent presence of benzoylecgonine in all samples (n = 21/21) collected in 2020, with a mean benzoylecgonine concentration of 1456.4 mg/d/1000inh. These findings suggest a notably higher consumption of cocaine within Valencia compared to the areas studied in South Wales.

### Effluent efficiency within WWTP-1 and WWTP-2

As benzoylecgonine was detected within both WWTPs, effluent samples were collected to determine whether the cleaning process significantly impacted the compounds detected and was successfully cleared before the water left the site. Both WWTPs collected spot samples on the same days as the composite samples to measure this. Table 3 below provides the crude composite result for benzoylecgonine and the effluent spot result for benzoylecgonine combined with the percentage difference.

**Table 3:** Results of effluent wastewater samples and the percentage difference of benzoylecgonine from crude results.

WWTP	Crude results (ng/mL)	Effluent result (ng/mL)	% Difference
1	3.01	1.35	55
1	3.01	0.74	75
1	2.00	0.72	64
1	1.59	0.65	59
1	2.29	0.58	75
1	1.22	0.15	87
1	4.01	0.34	92
1	1.94	0.00	100
1	3.11	0.98	68
1	1.50	0.72	52
1	2.13	1.13	47
2	4.47	0.20	95
2	1.15	0.33	71
2	3.55	0.51	86
2	1.73	0.70	60
2	4.74	0.86	82

The results conclude that the cleaning process of wastewater is having a positive impact on clearing the presence of benzoylecgonine. These wastewater treatment plants have an average of 73% clean-up rate with the highest being 100% and the lowest being 47%. Benzoylecgonine is known for being glutinous and tends to adhere to surfaces such as sewage pipes. Benzoylecgonine is a common compound detected for contamination within laboratory settings including in general circulation such as bank notes^27^. There is no correlation between the removal efficiency for higher concentrations of benzoylecgonine and lower concentrations of benzoylecgonine. WWTP-2 shows the least fluctuation with removal efficiency whereas WWTP-1 shows a greater variation. The average removal of 73%, is comparable with a previous study that looked at the removal efficiency of benzoylecgonine in the South of Italy where the average removal was 77.85%^28^.

### Qualitative analysis of WWTP-1 and WWTP-2

Owing to the dynamic nature of NPS substances, the targeted method is restricted to the compounds validated within the technique. A qualitative analysis was carried out by re-processing the already acquired crude and effluent samples using Sciex PeakView® software to determine if any medicinal compounds were present but not included in the targeted method.

These compounds were chosen because these analytes are within the 100 most prescribed medications in UK hospitals. Methadone and buprenorphine are not present in this list but are the most common medications prescribed to treat opioid abuse^29^. The results were determined by looking at each compound's peak height and area of the monoisotopic mass and corresponding fragment ion. The qualification work was done with two fragments. The SCIEX 5600 + QToF has a syringe injection infusion. Each qualitative drug (e.g., Methadone 310 m/z & 265 m/z) was infused directly onto the QToF to determine the fragmentation pattern using ToF MS. Once the fragmentation pattern was determined, the previous wastewater acquisition data was retrospectively processed to look for this fragmentation pattern using the PeakView software. This allowed a fragmentation pattern without having to add the compounds into the validated method. Providing qualification only and no quantitation.

As this is a qualitative approach an exact concentration cannot be determined, a determination of whether a compound is present or not present can be reported. In total, 10 crude samples selected at random were re-processed for these compounds. Table 4 provides a breakdown of the results.

**Table 4:** Results from the qualitative analysis of 10 crude samples using Peakview.

Sample number	Analytes present
17th Oct–WWTP-1	Buprenorphine, Codeine, Nordiazepam, Methadone
23rd Oct–WWTP-1	Buprenorphine, Codeine, Nordiazepam, Methadone
25th Oct–WWTP-1	Buprenorphine, Codeine, Methadone,
30th Oct–WWTP-1	Buprenorphine, Codeine, Methadone, Nordiazepam, Oxazepam
6th Nov–WWTP-1	Buprenorphine, Codeine, Methadone, Nordiazepam, Oxazepam
8th Nov–WWTP-1	Buprenorphine, Codeine, Methadone, Nordiazepam
30th Oct–WWTP-2	Buprenorphine, Codeine, Methadone, Nordiazepam, Oxazepam
6th Nov–WWTP-2	Buprenorphine, Codeine, Methadone
13th Nov–WWTP-2	Buprenorphine, Codeine, Methadone, Nordiazepam
14th Nov–WWTP-2	Buprenorphine, Codeine, Methadone, Nordiazepam

The results suggest that there is a presence of drug misuse towards medicinal substances within the wastewater samples. The UK has an ageing population which in turn could contribute to the increasing number of medicinal substances being consumed. Including the influx of new synthetic opioids and synthetic benzodiazepines within the population, these results correspond with the demand for medicinal products. Buprenorphine, codeine, and methadone are present in all samples. Nordiazepam and oxazepam are found in most samples. Diazepam was not chosen as a target analyte as diazepam is not commonly seen in urine and based on the metabolism of diazepam, the metabolites were investigated instead. Morphine was not detected in any of the samples, this could be explained by morphine being present in its conjugated form and with no hydrolysis present in this method, the free drug has not been released for detection. Effluent samples were also checked for the presence of these medicinal compounds. All samples were found to be negative.

### Hydrolysis

Another possibility for the minimal detection of NPS is that glucuronide compounds formed during metabolism are not broken down before analysis through a hydrolysis method. An experiment was designed to determine whether compounds were being missed due to their conjugated forms. Six wastewater samples were randomly selected for this experiment, each sample was separated into 5 individual aliquots of 25 mL along with the addition of β-glucuronidase enzyme BG Turbo was added to each aliquot. Samples were then placed into an incubator at 55 °C. Each sample included an internal standard allowing a qualitative check that extraction was successful.

The samples were left in an incubator, removed at a time interval (0, 1 h, 2 h, 24 h, and 48 h), analysed on the LC-ToF-MS method, and processed qualitatively using the Peakview® software. The results of the hydrolysis experiment concluded that there is an increase in compound concentration through hydrolysis, meaning that there is a change in concentration due to the breakdown of conjugated compounds.

Using BG Turbo as the enzyme, it was evident that most compounds reached complete hydrolysis at 2 h, the only difference was with THC-COOH and buprenorphine as hydrolysis was instant. The hydrolysis was measured based on an increase in peak height for all compounds. There were 6 compounds investigated, all being the more common medicinal substances consumed in the UK and compounds known to have glucuronide analogues: morphine-glucuronide, codeine-glucuronide, buprenorphine-glucuronide, oxazepam-glucuronide, nordiazepam-glucuronide, and THC-COOH-glucuronide.

One of the samples, showed an increase of 200% for codeine between pre- and post-hydrolysis, indicating that the glucuronide analogue was preventing detection for codeine before hydrolysis. Codeine was not detected before hydrolysis. Nordiazepam presented an increase of 173% and 172% in peak area from pre- to post-hydrolysis after 2 h. Table 5 illustrates the peak area results for pre- and post-hydrolysis.

**Table 5:** Results of pre- and post-hydrolysis with BG Turbo at 2 h incubation.

	Sample number & Peak area value Pre- and Post-hydrolysis											
	1		2		3		4		5		6	
	Pre-	Post-	Pre-	Post-	Pre-	Post-	Pre-	Post-	Pre-	Post-	Pre-	Post-
Morphine	–	–	7,003,117	13,381,175	–	–	0	556,751	525,954	942,339	337,852	827,902
Codeine	924,691	1,697,732	4,113,860	23,518,233	1,016,217	11,514,849	–	–	–	–	527,902	56,294,252
Nordiazepam	163,959	2,821,115	0	3,871,803	0	2,324,085	186,855	2,470,744	0	3,432,330	219,661	2,975,828
Buprenorphine	285,664	3,990,042	–	–	–	–	–	–	–	–	–	–
THC-COOH	–	–	0	316,335	0	919,742	–	–	–	–	–	–

It is well documented that these methods require hydrolysis when testing in urine. Stated in their study that hydrolysis should not be required due to in-sewer deconjugation and is normally removed by filtration and solid phase extraction as demonstrated by Gao et al.^30^ and Bijlsma et al.^31^. Another study looked at non-targeted SWATH for the identification of 47 SCRA compounds within the urine and used hydrolysis to ensure that the conjugate forms of the SCRAs were degraded to aid detection^32^. Based on this small experiment looking at glucuronide analogues of some of the more common medicinal substances, SPE does not optimally remove all analogues. Any additional concentrations of analytes may be missed if hydrolysis is not carried out on wastewater samples.

## Limitations

This study employs a robust methodology for detecting and quantifying illicit substances in wastewater; however, several limitations must be acknowledged to contextualise our findings accurately. Firstly, we opted to forego pre-filtration based on previous studies indicating potential analyte loss through this process. While pre-filtration can remove particulate matter that might interfere with solid-phase extraction (SPE) efficiency, avoiding it helps maintain the integrity of analyte concentrations. Although this choice might have led to lower detection rates due to potential blockage of SPE cartridges by impurities, the method's successful identification of substances like benzoylecgonine demonstrates its effectiveness.

Sampling was conducted exclusively on weekends, assuming peak drug consumption during these periods. This approach aimed to efficiently utilise resources while targeting high-drug usage times, which are critical for understanding significant usage patterns. However, this strategy might not capture the full spectrum of drug consumption throughout the week. While weekday sampling could provide a more comprehensive view, our methodology focused on highlighting peak consumption trends and multiple previous studies have reported higher concentrations of illicit substances detected over weekends rather than weekdays^33,34^. Additionally, the targeted analysis approach, focusing on a pre-defined list of compounds, ensured high accuracy and sensitivity but inherently limited the detection of emerging new psychoactive substances (NPS). Despite this, the targeted method provided reliable data on established substances, offering crucial insights into prevalent drug use trends. The study may underestimate the overall presence and usage of NPS in the studied area, as new and unidentified NPS could be flowing through the wastewater system undetected.

Differences in population size and wastewater flow rates significantly impact the estimates of drug consumption derived from wastewater analysis. Larger populations generate more wastewater, leading to higher concentrations of drug residues, whereas smaller populations might dilute these concentrations, potentially underestimating actual drug usage. This variability necessitates the normalisation of data per capita to standardise results and make meaningful comparisons, although inherent variability remains^35,36^. Daily and seasonal variations in flow rates further contribute to fluctuations in drug concentration measurements. Higher flow rates tend to dilute drug residues, while lower flow rates concentrate them. Additionally, the stability of drug residues can vary with flow rates, influencing their detection. Adjusting for flow rate variations by calculating mass loads (mg/day) provides more accurate estimates of the total drug load entering the WWTP, but variability remains due to factors such as sampling times and locations, and in-sewer degradation, which affects the stability of drug metabolites^35,37^. Studies have shown that while normalising data for these factors helps mitigate their impact, these adjustments cannot eliminate the variability. For example, dynamic population normalisation has been used to improve the accuracy of wastewater-based epidemiology (WBE) by accounting for fluctuations in the population served by WWTPs, as static estimates often fail to capture transient changes in population size^35,36^.

Comprehensive sampling strategies and robust analytical methods are essential to accurately assess and compare drug use patterns across different regions and periods. The use of dynamic population estimates and careful consideration of flow rates can enhance the reliability of WBE data, although challenges remain in completely mitigating these sources of variability. The integration of qualitative analysis alongside targeted methods broadens the detection scope, providing valuable insights into additional substances without exact concentration measurements. While the absence of detected NPS may reflect limitations in the detection method or the dynamic nature of NPS, the inclusion of qualitative analysis demonstrates the method's effectiveness. Despite these limitations, the study's methodological choices were carefully considered to provide accurate and meaningful data within the study design constraints.

## Conclusion

Our study's analysis of wastewater samples revealed an absence of New Psychoactive Substances (NPS) in the influents of two wastewater treatment plants (WWTPs) in South Wales. However, the presence of benzoylecgonine was detected in the influents of both WWTPs, suggesting a significant finding. This observation indicates that either NPS consumption is minimal within this population area or that the dosage of NPS is notably lower compared to commonly abused medicinal drugs like cocaine or cannabis. Quantitatively, the average benzoylecgonine clean-up rate at the WWTPs was calculated to be 73%, with a range spanning from 47 to 100%. This variance in clean-up efficiency between the two WWTPs suggests irregularities in the treatment processes. Surprisingly, there is no discernible correlation between higher benzoylecgonine concentrations and lower clean-up efficiency. Such quantitative analysis provides insights into wastewater treatment processes' efficacy and highlights improvement areas. This phenomenon could be attributed to various factors. Firstly, the type of NPS in common use might have evolved, rendering the target analytes obsolete. Alternatively, the concentration of NPS might be too diluted in water to be reliably detected. Additionally, the uptake and popularity of new NPS within a population area might remain low until they become well-recognised, contributing to the sporadic presence of these substances. Furthermore, our findings suggest that while many NPS analytes dissipate rapidly from circulation, some manage to establish themselves within the population over time. This underscores the importance of continuous monitoring and adaptation of detection methods to stay ahead of emerging trends in substance use. By quantitatively analysing the clean-up rates and correlating them with substance concentrations, we can better understand the dynamics of substance usage patterns within communities and tailor interventions accordingly.

### Supplementary Information

Supplementary Figure S1.

Supplementary Table S1.

Supplementary Table S2.

## Data Availability

The datasets generated and/or analysed during the current study are not publicly available due to privacy but are available from the corresponding author upon reasonable request.
