# Supplementary figures and images for: Analysis of crude wastewater from two treatment plants in South Wales for 35 new psychoactive substances and cocaine, and cannabis
Source: Sci Rep. 2024 Aug 29;14:20129. doi: 10.1038/s41598-024-70378-7 (PMC11362326; doi:10.1038/s41598-024-70378-7)

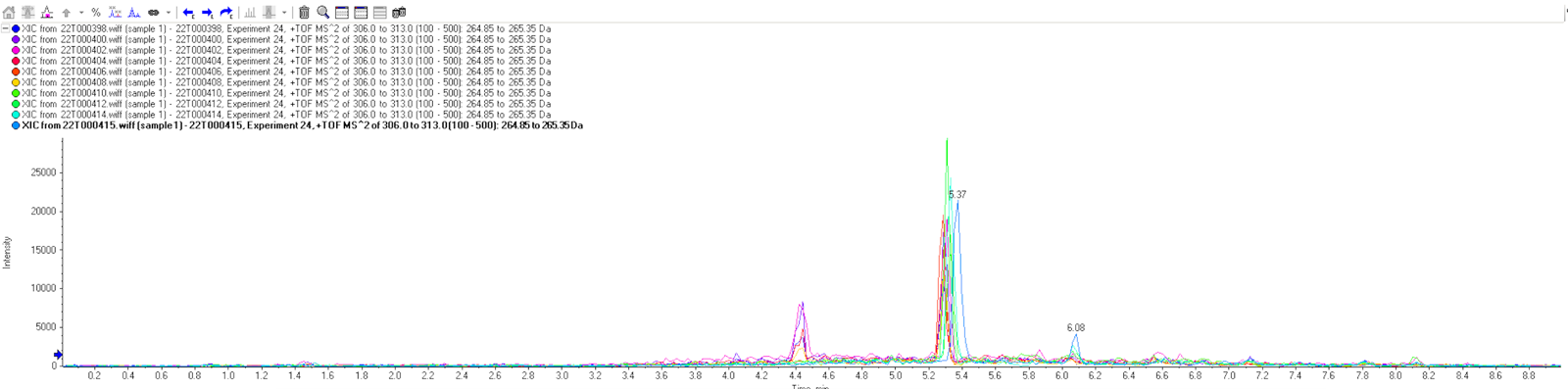


Figure S1 Image from Peakview illustrating the presence of Methadone in each sample

Supplement: Supplementary file 1 — Supplementary Figure S1. [file 41598_2024_70378_MOESM1_ESM.docx]
