# Supplementary material for: Analysis of crude wastewater from two treatment plants in South Wales for 35 new psychoactive substances and cocaine, and cannabis
Source: Sci Rep. 2024 Aug 29;14:20129. doi: 10.1038/s41598-024-70378-7 (PMC11362326; doi:10.1038/s41598-024-70378-7)
Supplement: Supplementary file 2 — Supplementary Table S1. [file 41598_2024_70378_MOESM2_ESM.docx]

Table S1 Table illustrating the retention time, SWATH window, precursor ion and fragment ion for all target compounds

| Analyte | Retention Time | SWATH Window | Monoisotopic Mass (Da) | Precursor Ion (m/z) | Fragment Ion (m/z) |
| --- | --- | --- | --- | --- | --- |
| Alprazolam | 5.92 | 1 | 308.082 | 309.061 | 281.055 |
| Benzoylecgonine | 4.44 | 1 | 289.131 | 290.091 | 168.078 |
| Benzyl Piperazine | 1.54 | 1 | 176.131 | 177.055 | - |
| Fentanyl | 4.80 | 1 | 336.220 | 337.206 | 188.107 |
| Alprazolam-D5 | 5.91 | 1 | 313.805 | 314.106 | 256.009 |
| JWH-018-D5 | 5.34 | 1 | 376.512 | 377.985 | 154.956 |
| Fentanyl-D5 | 4.83 | 1 | 341.501 | 342.004 | 187.912 |
| 25C-NBOMe-D3 | 5.09 | 1 | 338.847 | 339.959 | 123.874 |
| LSD-D3 | 4.67 | 1 | 326.458 | 327.042 | 281.655 |
| UR-144 5-hydroxypentyl-D5 | 6.62 | 1 | 332.498 | 333.001 | - |
| TFMPP-D4 | 4.60 | 1 | 234.256 | 235.852 | 189.666 |
| 25I-NBOMe-D3 | 5.32 | 1 | 430.258 | 431.902 | 123.845 |
| Benzoylecgonine-D3 | 4.60 | 1 | 292.344 | 293.992 | 170.822 |
| Benzylpiperazine-D7 | 1.18 | 1 | 183.301 | 184.292 | - |
| Mephedrone | 4.16 | 2 | 177.115 | 178.118 | 160.107 |
| 4-Methylethcathinone | 4.28 | 4 | 191.131 | 192.137 | 174.125 |
| Methylone | 3.91 | 7 | 207.089 | 208.082 | 160.061 |
| Norfentanyl | 4.30 | 11 | 232.157 | 233.103 | 85.0806 |
| PB-22 3-carboxyindole | 4.58 | 11 | 231.310 | 232.134 | 188.148 |
| TFMPP | 4.59 | 11 | 230.103 | 231.096 | 188.054 |
| 5F-PB-22 3-carboxyindole | 5.75 | 14 | 249.281 | 250.125 | 162.052 |
| Methoxetamine | 4.48 | 14 | 247.157 | 248.164 | 203.103 |
| 2C-B | 4.54 | 16 | 260.135 | 261.015 | 242.985 |
| 5-MeO-DALT | 4.47 | 18 | 270.173 | 271.172 | 174.091 |
| MDPV | 4.59 | 18 | 275.152 | 276.163 | 126.102 |
| LSD | 4.64 | 27 | 323.199 | 324.217 | 223.124 |
| UR-144 4-Hydroxypentyl | 6.63 | 27 | 327.584 | 328.227 | 125.094 |
| UR-144 5-Hydroxypentyl | 6.63 | 27 | 327.584 | 328.227 | 125.094 |
| 25C-NBOMe | 5.04 | 28 | 335.128 | 336.139 | 121.061 |
| AB-PINACA | 6.39 | 28 | 330.205 | 331.971 | 98.9005 |
| Etizolam | 6.00 | 30 | 342.070 | 343.075 | 314.002 |
| THC-COOH | 5.99 | 30 | 344.198 | 345.152 | 327.141 |
| UR-144 COOH | 6.60 | 30 | 341.448 | 343.303 | 240.235 |
| 5F-AB-PINACA | 5.89 | 31 | 348.196 | 349.204 | 304.186 |
| 2-OXO-LSD | 4.07 | 32 | 355.432 | 356.195 | 237.108 |
| 5F-MDMB-PINACA (5F-ADB) | 6.49 | 33 | 377.510 | 378.201 | 318.207 |
| AB-PINACA pentanoic acid | 5.17 | 33 | 360.411 | 361.195 | 344.165 |
| AB-FUBINACA metabolite | 6.13 | 34 | 369.394 | 370.159 | 324.156 |
| JWH-018 pentanoic acid | 6.79 | 34 | 371.164 | 372.157 | 155.041 |
| MDMB-CHMICA O-desmethyl acid | 5.89 | 34 | 370.544 | 371.204 | 240.144 |
| AM2201 4-Hydroxypentyl | 6.62 | 35 | 375.478 | 376.178 | 155.055 |
| APICA 4-hydroxypentyl | 7.08 | 36 | 381.577 | 382.236 | 135.194 |
| APINACA 4-hydroxypentyl | 7.08 | 36 | 381.526 | 382.252 | 135.115 |
| APINACA 5-hydroxypentyl | 7.08 | 36 | 381.526 | 382.252 | 135.115 |
| 5F-APICA 4-hyroxypentyl (STS-135) | 6.95 | 39 | 398.514 | 399.245 | 135.117 |
| 5F-APINACA 4-hydroxypentyl | 6.97 | 39 | 399.510 | 400.231 | 135.114 |
| 25I-NBOMe | 5.25 | 44 | 427.064 | 428.073 | 121.066 |
