# Supplementary material for: Analysis of crude wastewater from two treatment plants in South Wales for 35 new psychoactive substances and cocaine, and cannabis
Source: Sci Rep. 2024 Aug 29;14:20129. doi: 10.1038/s41598-024-70378-7 (PMC11362326; doi:10.1038/s41598-024-70378-7)
Supplement: Supplementary file 3 — Supplementary Table S2. [file 41598_2024_70378_MOESM3_ESM.docx]

Table S2 Table illustrates the results of the recovery, accuracy, and precision analysis for each target compound.

| Analyte | Recovery (%) | Accuracy (%) | | | Precision (%) | | | | | |
| --- | --- | --- | --- | --- | --- | --- | --- | --- | --- | --- |
|  |  |  |  |  | Intra-day | | | Inter-day | | |
|  |  | Low | Mid | High | Low | Mid | High | Low | Mid | High |
| 25C-NBOMe | 109% | 100 | 86 | 84 | 18 | 12 | 8 | 19 | 16 | 12 |
| 25I-NBOMe | 111% | 89 | 81 | 91 | 16 | 12 | 14 | 19 | 13 | 17 |
| 2C-B | 131% | 100 | 93 | 87 | 20 | 18 | 17 | 28 | 26 | 20 |
| 2-OXO-LSD | 100% | 89 | 86 | 91 | 15 | 19 | 19 | 20 | 20 | 22 |
| 4-Methylethcathinone | 106% | 89 | 94 | 97 | 16 | 19 | 19 | 22 | 30 | 22 |
| 5F-AB-PINACA | 105% | 89 | 88 | 94 | 18 | 17 | 16 | 20 | 18 | 17 |
| 5F-APICA | 94% | 89 | 86 | 96 | 18 | 19 | 17 | 20 | 20 | 23 |
| 5F-APINACA | 102% | 100 | 81 | 77 | 16 | 18 | 15 | 20 | 19 | 25 |
| 5F-MDMB-PINACA | 108% | 89 | 97 | 99 | 18 | 17 | 14 | 19 | 18 | 16 |
| 5F-PB-22 | 104% | 100 | 83 | 86 | 19 | 20 | 17 | 20 | 25 | 20 |
| 5-MeO-DALT | 109% | 99 | 97 | 99 | 14 | 12 | 16 | 18 | 13 | 18 |
| AB-FUBINACA | 103% | 89 | 100 | 97 | 18 | 17 | 13 | 18 | 19 | 15 |
| AB-PINACA | 90% | 100 | 97 | 96 | 20 | 16 | 10 | 22 | 17 | 12 |
| AB-PINACA Metabolite | 81% | 100 | 88 | 97 | 18 | 18 | 12 | 19 | 24 | 13 |
| Alprazolam | 103% | 99 | 86 | 94 | 18 | 18 | 18 | 20 | 22 | 24 |
| AM2201 4-Hydroxypentyl | 103% | 100 | 83 | 87 | 17 | 19 | 16 | 24 | 22 | 18 |
| APICA 4-hydroxypentyl | 112% | 89 | 91 | 92 | 18 | 19 | 19 | 19 | 21 | 21 |
| APINACA 4-hydroxypentyl | 112% | 89 | 96 | 96 | 20 | 19 | 14 | 22 | 21 | 16 |
| APINACA 5-hydroxypentyl | 112% | 89 | 96 | 96 | 20 | 19 | 14 | 22 | 21 | 16 |
| Benzoylecgonine | 106% | 80 | 77 | 96 | 15 | 20 | 16 | 21 | 29 | 20 |
| Benzyl Piperazine | 118% | 100 | 97 | 89 | 20 | 16 | 19 | 25 | 21 | 23 |
| Etizolam | 88% | 100 | 91 | 96 | 17 | 11 | 15 | 18 | 14 | 17 |
| Fentanyl | 94% | 89 | 91 | 99 | 20 | 17 | 9 | 22 | 20 | 10 |
| JWH-018 Pentanoic Acid | 102% | 89 | 94 | 98 | 16 | 18 | 19 | 17 | 22 | 21 |
| LSD | 98% | 89 | 79 | 99 | 19 | 16 | 19 | 23 | 7 | 23 |
| MDMB-CHMICA | 108% | 100 | 94 | 95 | 18 | 17 | 18 | 19 | 19 | 20 |
| MDPV | 92% | 100 | 97 | 82 | 17 | 16 | 20 | 23 | 17 | 24 |
| Mephedrone | 105% | 100 | 97 | 98 | 16 | 18 | 17 | 19 | 16 | 24 |
| Methoxetamine | 108% | 89 | 88 | 89 | 13 | 19 | 16 | 14 | 19 | 21 |
| Methylone | 85% | 89 | 100 | 98 | 19 | 10 | 11 | 28 | 10 | 10 |
| Norfentanyl | 67% | 100 | 91 | 86 | 19 | 12 | 19 | 22 | 14 | 23 |
| PB-22 Carboxyindole | 53% | 100 | 100 | 99 | 17 | 18 | 18 | 18 | 20 | 22 |
| TFMPP | 96% | 89 | 100 | 100 | 16 | 17 | 10 | 17 | 21 | 12 |
| THC-COOH | 103% | 94 | 100 | 89 | 16 | 15 | 16 | 24 | 24 | 21 |
| UR-144 4-Hydroxypentyl | 103% | 89 | 91 | 95 | 20 | 11 | 16 | 24 | 13 | 21 |
| UR-144 5-Hydroxypentyl | 103% | 89 | 91 | 95 | 20 | 11 | 16 | 24 | 13 | 21 |
| UR-144 COOH | 96% | 94 | 100 | 89 | 18 | 7 | 15 | 21 | 25 | 19 |
